# Displaced bronchus and anomalous pulmonary vein passing dorsal to the pulmonary artery in a patient who underwent right upper lobectomy for lung cancer with lymph node metastases: a case report

**DOI:** 10.1186/s44215-022-00014-1

**Published:** 2023-01-09

**Authors:** Hayato Konno, Mitsuhiro Isaka, Tetsuya Mizuno, Hideaki Kojima, Yasuhisa Ohde

**Affiliations:** Division of Thoracic Surgery, Shizuoka Cancer Center, 1007 Shimonagakubo, Nagaizumi-cho Sunto-gun, Shizuoka, 411-8777 Japan

**Keywords:** Displaced bronchus, Anomalous pulmonary vein, Non-small-cell lung cancer, Lymph node dissection

## Abstract

**Background:**

Tracheobronchial anomalies are extremely rare and are often associated with pulmonary arteriovenous anatomical anomalies. An anomalous right upper vein segment that passes between the pulmonary artery (PA) and bronchus is a rare vascular abnormality. We report a case of a displaced superior posterior branch (B2) that was independent of the superior apical/anterior branch (B1 + 3) accompanied by anomalous right superior pulmonary vein (SPV) anatomy in a patient who underwent right upper lobectomy for lung cancer with lymph node metastases.

**Case presentation:**

A 73-year-old asymptomatic woman was shown to have an abnormal shadow on chest radiography performed during medical checkup and visited our hospital for further evaluation. The patient was diagnosed with primary lung adenocarcinoma (c-T1cN1M0, stage IIB) involving the right superior posterior segment (S2) with an abnormally displaced B2 and an anomalous upper vein segment that was observed to run dorsal to the PA and anterior to the right upper bronchus. We performed right upper lobectomy and systematic hilar and mediastinal nodal dissection via video-assisted thoracoscopic surgery. Intraoperative findings revealed a displaced B2 bronchus independent from the B1 + 3 and an anomalous SPV, which was consistent with preoperative imaging findings.

**Conclusion:**

Preoperative bronchoscopy and three-dimensional computed tomography angiography (3D-CTA) are essential to confirm bronchial bifurcation and vascular abnormalities to aid with meticulous surgical planning to select the optimal operative technique.

**Supplementary Information:**

The online version contains supplementary material available at 10.1186/s44215-022-00014-1.

## Background

Tracheobronchial anomalies are extremely rare, with a reported prevalence of 0.1–2% in the general population [1–5], and are often associated with pulmonary arteriovenous anatomical anomalies; therefore, close and careful evaluation is warranted in suspected cases [4]. An anomalous right upper vein segment that passes between the pulmonary artery (PA) and bronchus is a rare vascular abnormality [6]. We report a case of a displaced superior posterior branch (B2) that was independent of the superior apical/anterior branch (B1 + 3) accompanied by anomalous right superior pulmonary vein (SPV) anatomy in a patient who underwent right upper lobectomy for lung cancer with lymph node metastases. We have additionally presented a literature review.

## Case presentation

**Fig. 1:**
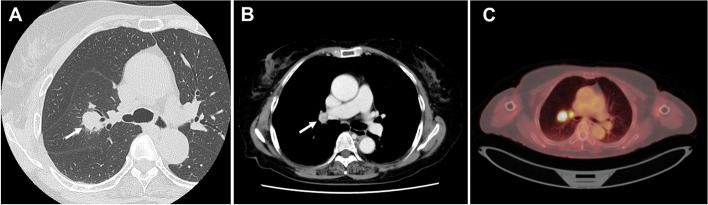
**A** Chest CT scan using the lung window setting showing a pulmonary nodule measuring 23 mm in diameter in the right upper lobe (arrow). **B** Contrast-enhanced CT scan using the mediastinal window setting showing hilar lymph node (station 11s) enlargement (arrow). **C** PET/CT scan showing 18F-FDG uptake in the tumor and in the hilar lymph nodes (station 11s). CT: computed tomography, PET: positron emission tomography, 18F-FDG: 18-fluorine-fluorodeoxyglucose

**Fig. 2:**
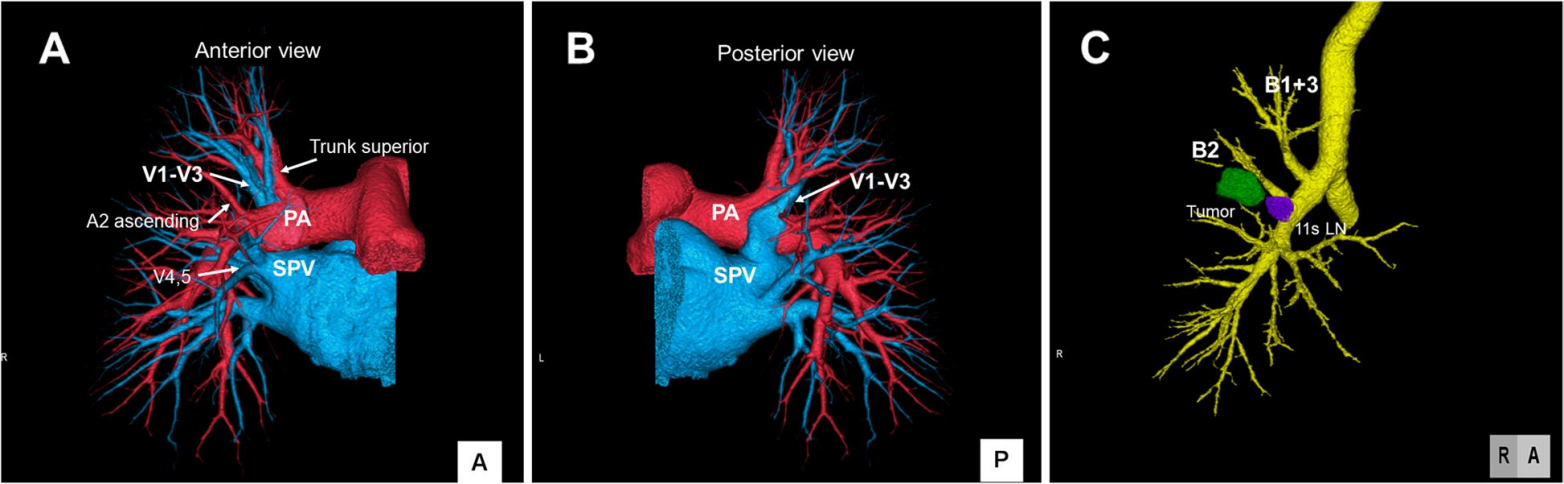
**A** A 3D-CTA scan (anterior view) showing the V1–V3 segment passing dorsal to the right main PA. **B** A 3D-CTA scan (posterior view) showing the V1–V3 segment draining into the SPV. **C** The B2 and B1 + 3 segments are observed to branch separately from the right main bronchus. The tumors are marked green and the station 11s lymph nodes appear purple. LN: lymph node, PA: pulmonary artery, SPV: superior pulmonary vein, 3D-CTA: three-dimensional computed tomography angiography

A 73-year-old asymptomatic woman was shown to have an abnormal shadow on chest radiography during a medical checkup and presented to our hospital for further evaluation. Computed tomography (CT) revealed a solid nodule (23 mm) in the right upper lobe (S2) (Fig. 1A) and hilar lymph node (station 11s) enlargement (Fig. 1B). Notably, 18-fluorine-fluorodeoxyglucose positron emission tomography/computed tomography (18F-FDG PET/CT) revealed FDG accumulation in the nodule (maximum standardized uptake value 27.1) and in the station 11s lymph nodes (maximum standardized uptake value 5.79) (Fig. 1C). Contrast-enhanced three-dimensional CT angiography (3D-CTA) revealed the V1–V3 segment of the right upper lobe PV passed dorsal to the right main PA and anterior to the right upper bronchus (RUB), and the B2 and B1 + 3 segments originated as separate branches from the right main bronchus (Fig. 2). Systemic CT tomography revealed no other tumors. Bronchoscopy revealed displaced anomalous B2 and B1 + 3 segments that originated from the right main bronchus, and transbronchial lung biopsy of a specimen obtained from the displaced bronchus confirmed diagnosis of adenocarcinoma. Therefore, the patient was diagnosed with primary lung adenocarcinoma (c-T1cN1M0, stage IIB) involving the right S2 with a displaced abnormal bronchus and anomalous upper vein segment that ran dorsal to the PA and anterior to the RUB.

**Fig. 3:**
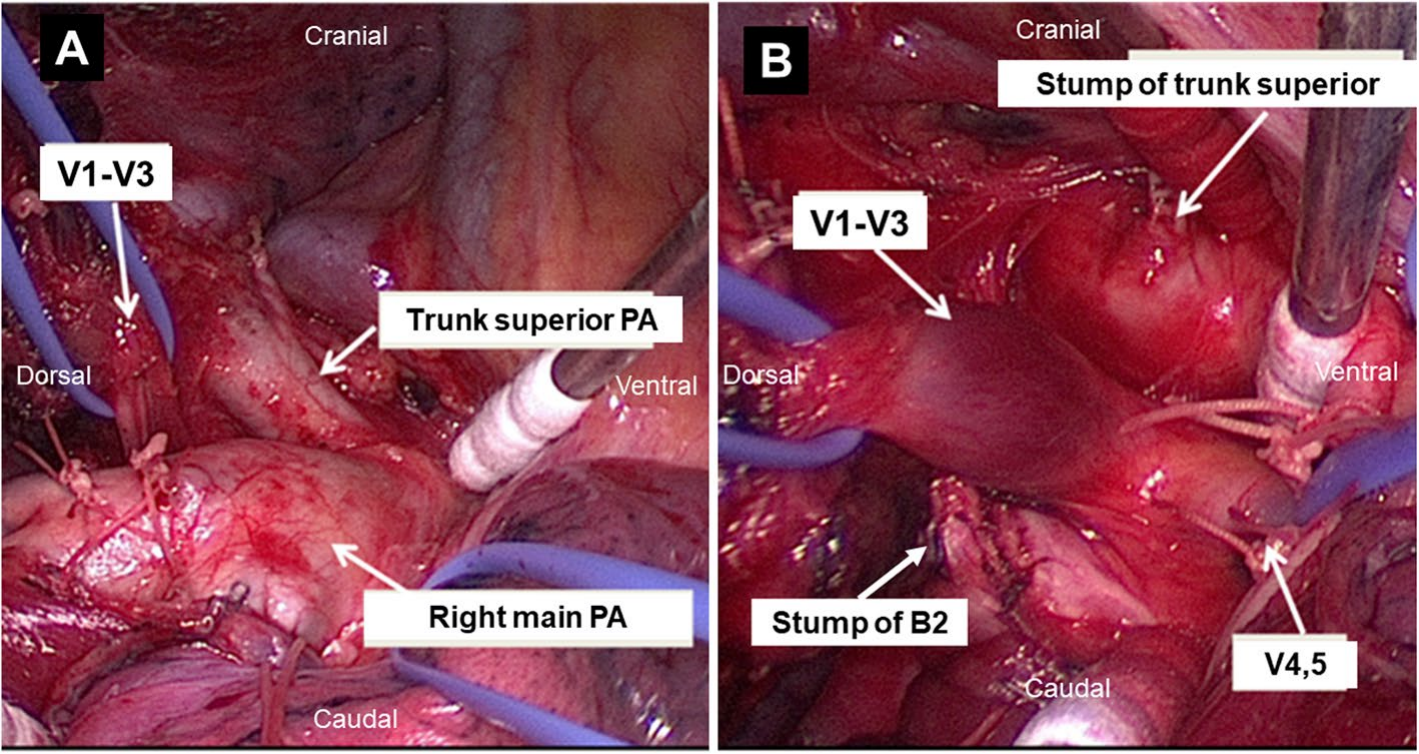
**A** Intraoperative thoracoscopic findings showing the V1–V3 segment passing dorsal to the right main PA. **B** The superior trunk of the PA (A1 + 3) and displaced B2 segment are divided and the anomalous V1–V3 are carefully exposed via deployment of the main PA. PA: pulmonary artery

We performed right upper lobectomy via video-assisted thoracoscopic surgery using an 8 cm access window. Intraoperatively, we observed an incomplete fissure between the upper and middle lobes along with an anatomical variation in the V1–V3 segment, which passed dorsal to the right main PA and drained into the SPV (Fig. 3A). The interlobar fissure between the upper and middle lobes was divided using an endostapler. The ascending A2 segment was ligated and cut followed by V1–3 dissected and encircled. The main PA was exposed from the ventral pulmonary hilum, and then A1 + 3 was dissected and divided using an endostapler. Hilar lymph nodes were carefully dissected, and displaced B2 was divided using an endostapler prior to V1–3 (Additional file 1: Supplementary Figure S1). The processing of B2 prior to V1–3 provided a good dorsal view, and we identified the abnormal V1–V3 segment posterior to the right main PA and sufficiently exposed to the left atrium side (Fig. 3B). The abnormal V1–V3 was divided using an endostapler. The B1 + 3 segment was divided using an endostapler, and the right upper lobe was removed, and we carefully dissected the hilar and mediastinal lymph nodes.

The patient’s postoperative course was uneventful. Histopathological evaluation confirmed diagnosis of acinar adenocarcinoma (maximal diameter 22 mm) and station 11s node metastases (p-T2aN1M0, stage IIB). Metastatic cancer was detected in one of the 11 dissected lymph nodes. The patient was administered cisplatin and vinorelbine as adjuvant chemotherapy. The patient is recurrence free, 4 years postoperatively.

## Discussion

Tracheobronchial anomalies are classified as supernumerary or displaced bronchi [1]. Reportedly, the incidence of tracheobronchial anomalies is 0.35–0.76%, and the right upper lobe (75–89%) is most commonly affected [2–4]. A tracheal bronchus includes a variety of bronchial anomalies associated with the trachea or the main bronchus and is typically observed in the upper lobes [3]. Based on Ohta’s classification, the anatomy in our patient was classified as “displaced segmental bronchus (III-B: B1 + 3 from the main bronchus).” Although this variant may be considered a branching of the B2 segment from the intermediate bronchus trunk, Ohta’s classification typically categorizes the displaced segmental bronchus from the intermediate bronchus as a B6 branching abnormality (III-C). In contrast, based on Yaginuma’s classification, this anomaly is classified as “right B2 type,” (7 of 6480 cases, prevalence 0.1%), which is extremely rare.

Tracheobronchial anomalies are often associated with pulmonary arteriovenous anatomical anomalies, and careful evaluation is warranted in suspected cases. Shiina et al. reported that variant-type PV anomalies occur more commonly in the right lung (32.8% of all PV anomalies) than in the left lung (2.6%) [7]. Since the first case of an anomalous PV passing posterior to the intermediate bronchus reported in 1984 [8], studies have described several types of PV anomalies [6, 9, 10]. Akiba et al. described six types of variants of the right SPV as a new classification based on the route and inflow site [6]. Based on Akiba’s classification, this anomaly is classified as a “variant other pulmonary vein type,” in which the anomalous right SPV passes through sites other than posterior to the intermediate bronchus and drains into the PV. Several studies have reported absence of the right SPV at the anterior hilum with an anomalous V1–V3 segment that passes between the PA and the bronchus [11–17].

To our knowledge, only eight studies have reported right upper lobe resection for lung cancer in patients with concomitant ectopic variation of the right SPV and RUB (Table 1) [11, 12, 14, 18–22].. The site of the right SPV and the RUB in our patient differed from sites observed in patients described by previous studies. Only one previous study has reported the type of anomalous right SPV and RUB observed in the present case [15]. However, the patient had right lower lobe lung cancer. No report has described right upper lobectomy for lung cancer. To date, most researchers have focused on early-stage cancers, and in addition to our present study, only one previous study has discussed lung cancer with lymph node metastasis [18].

**Table 1:** Previous reports of right upper resection for lung cancer in patients with displaced bronchus and anomalous pulmonary vein

Author (year)	Age/sex	Displaced bronchus	Anomalous PV	LNM/Stage	Surgical procedure
Yurugi Y (2012) [11]	58/F	B1 directly from trachea (TB)	V1–V3; dorsally PA, draining LA	Negative/IA	Lobectomy
Xu XF (2014) [12]	39/F	B2 directly from trachea (TB)	V2; dorsally PA, draining LA	Negative/IA	Segmentectomy
Tajima K (2016) [18]	69/F	B1 from main bronchus independently from B2 + 3 (B1 type)	V2; dorsally intermediate bronchus, draining LA	Positive/IIIA	Lobectomy
Nakamura Y (2017) [14]	66/F	B1 directly from trachea (TB)	V1–V3; dorsally PA, draining SPV	NM	Lobectomy
Kawasaki H (2017) [19]	45/M	RUB directly from trachea (TB)	PAPVC (V1–V3 draining SVC)	Negative/IA	Lobectomy
Qi W (2020) [20]	51/F	B1 + 2 directly from trachea (TB)	V2; dorsally PA, draining SPV	Negative/IA	Segmentectomy
Sugiura Y (2021) [21]	73/M	B1 directly from trachea (TB)	PAPVC (V1–V3 draining SVC)	Negative/IA	Lobectomy
Momose N (2021) [22]	73/F	B2 from main bronchus independently from B1 + 3 (B2 type)	V2; dorsally intermediate bronchus, draining V6 (IPV)	Negative/IA	Lobectomy
Present case	73/F	B2 from main bronchus independently from B1 + 3 (B2 type)	V1-V3; dorsally PA, draining SPV	Positive/IIB	Lobectomy

*IPV* inferior pulmonary vein, *LA* left atrium, *LNM* lymph node metastasis, *NM* not mentioned, *PA* pulmonary artery, *PAPVC* partial anomalous pulmonary venous connection, *PV* pulmonary vein, *SPV* superior pulmonary vein, *SVC* superior vena cava, *TB* tracheal bronchus

We report an extremely rare type of life-threatening right SPV anomaly; the right SPV passed anterior to the right main PA. Therefore, the space between the right main PA and the right main bronchus was considered an area that did not contain any blood vessel. However, the findings in the current case disproved this theory. Notably, 3D-CTA enables rapid and easy diagnosis of anomalous PVs, and its use should be encouraged for surgical planning to avoid massive intraoperative bleeding in patients who undergo anatomical resection [23, 24]. Previous studies have reported post-lobectomy cerebral infarction, which is attributable to the longer stump of the PV, with consequent stasis of blood flow and a high risk of thrombosis [25, 26]. Therefore, PV ligation and division should be carefully performed. In our case, we took care to ensure that the PV stump length was not overly long through sufficient exposure of V1–3 to the left atrium side.

Lymph node dissection is an important component of surgery performed for lung cancer with a displaced bronchus; however, limited data are available regarding lymph node dissection because lymphatic flow has not been widely investigated in displaced bronchi and may be associated with lung lobulation abnormalities. Some studies have reported segmentectomy for lung cancer with displaced bronchi [12, 20]; however, an individualized surgical approach and meticulous preoperative evaluation are warranted. Our patient had lymph node metastasis and underwent systematic nodal dissection; metastatic lymph nodes located between the displaced B2 and the intermediate bronchus were considered station 11s nodes.

In conclusion, we report a rare case of right upper lung cancer concomitant with an ectopic variation of the right SPV and RUB. Preoperative bronchoscopy and 3D-CTA are essential to confirm bronchial bifurcation and vascular abnormalities to aid with meticulous surgical planning to select the optimal operative technique.

## Supplementary Information

**Additional file 1: Supplementary Figure S1**. A 3D-CTA scan (posterior view) showing the location of displaced B2 and V1–V3 segment.

## Data Availability

All data for this study are presented in the manuscript. A part of the datasets is available from the corresponding author on reasonable request.

## Abbreviations

CT: Computed tomography
PA: Pulmonary artery
PET/CT: Positron-emission tomography/computed tomography
RUB: Right upper bronchus
SPV: Superior pulmonary vein
3D-CTA: Three-dimensional computed tomography angiography
