# Supplementary figures and images for: Displaced bronchus and anomalous pulmonary vein passing dorsal to the pulmonary artery in a patient who underwent right upper lobectomy for lung cancer with lymph node metastases: a case report
Source: Gen Thorac Cardiovasc Surg Cases. 2023 Jan 9;2:2. doi: 10.1186/s44215-022-00014-1 (PMC11533443; doi:10.1186/s44215-022-00014-1)

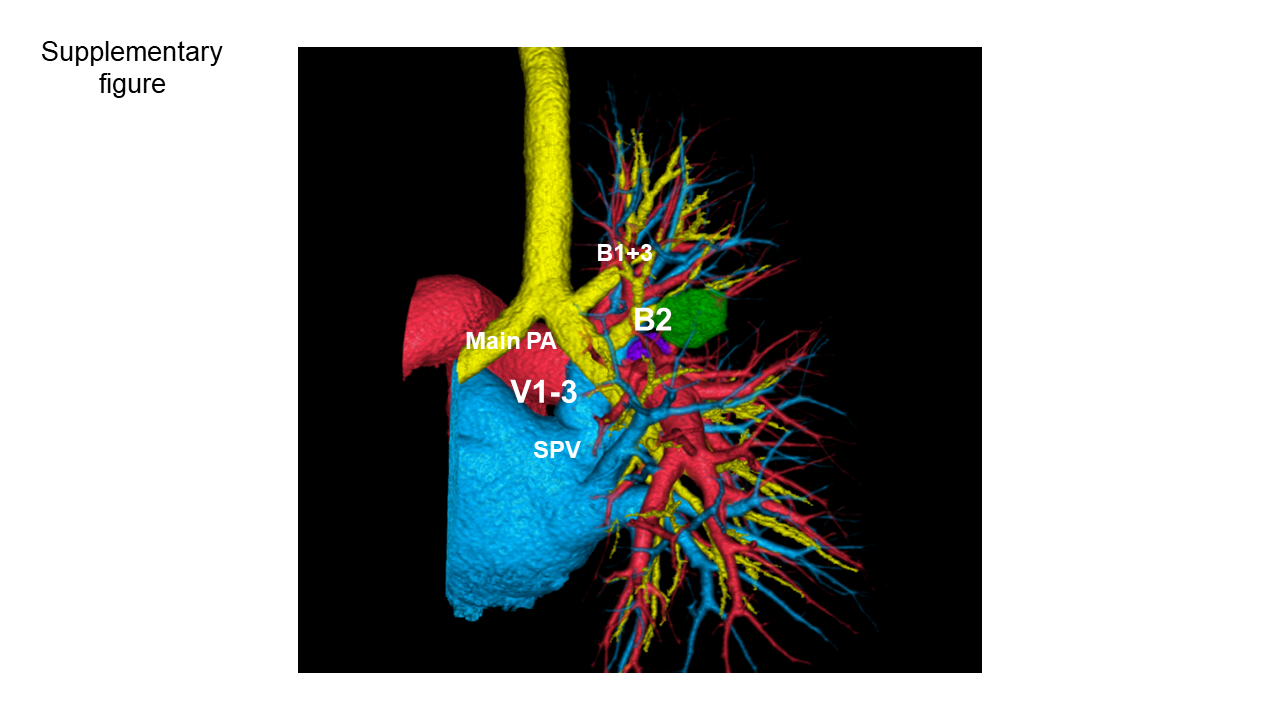

Supplement: Supplementary file 1 — Additional file 1: Supplementary Figure S1. A 3D-CTA scan (posterior view) showing the location of displaced B2 and V1–V3 segment. [file 44215_2022_14_MOESM1_ESM.tif]
